# 7-Pivaloyl-5,6-dihydro-4*H*-naphtho[3,2,1-*de*]isoquinoline-4,6-dione

**DOI:** 10.1107/S1600536810008378

**Published:** 2010-03-13

**Authors:** Hai-Tao Yu, Yi Wei, Yan Zhang

**Affiliations:** a School of Chemistry and Chemical Engineering, Nanjing University, Nanjing 210093, People’s Republic of China

## Abstract

In the crystal structure of the title compound, C_21_H_17_NO_3_, the dibenzo–isoquinoline–dione unit has a planar structure, the maximum atomic deviation being 0.091 (3) Å. The crystal structure is stabilized by π–π stacking [centroid–centroid distance = 3.851 (2) Å] and inter­molecular N—H⋯O hydrogen bonding.

## Related literature

The title compound is an azonafide analogue. For the bio­logical activity of 1,3,4(2*H*)-isoquinoline­trione derivatives, see: Malamas *et al.* (1994 ▶); Hall *et al.* (1994 ▶). For the anti­tumor properties of azonafide and analogues, see: Sami *et al.* (2000 ▶); Hutchings *et al.* (1988 ▶). For the synthesis, see: Zhang *et al.* (2000 ▶).
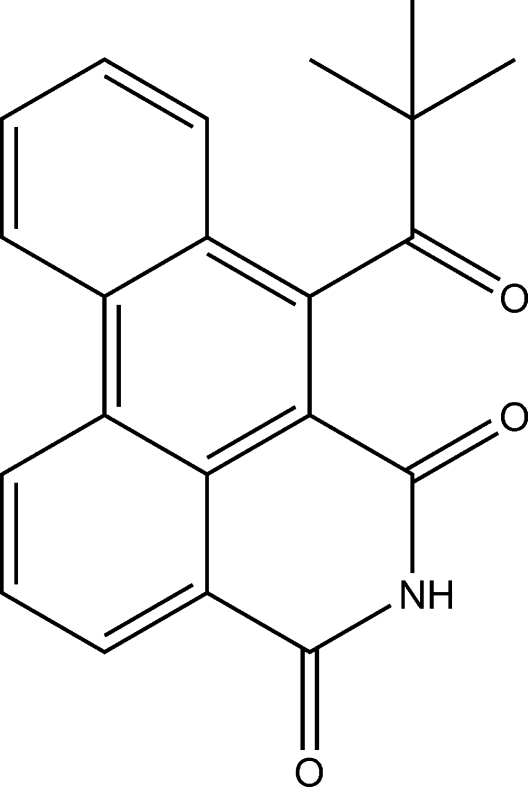


## Experimental

### 

#### Crystal data

C_21_H_17_NO_3_
*M* *_r_* = 331.36
Monoclinic, 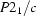

*a* = 11.569 (2) Å
*b* = 9.1150 (18) Å
*c* = 15.746 (3) Å
β = 101.12 (3)°
*V* = 1629.3 (6) Å^3^
*Z* = 4
Mo *K*α radiation
μ = 0.09 mm^−1^
*T* = 298 K
0.10 × 0.10 × 0.05 mm

#### Data collection

Enraf–Nonius CAD-4 diffractometer
Absorption correction: ψ scan (*CAD-4 EXPRESS*; Enraf–Nonius, 1994 ▶) *T*_min_ = 0.991, *T*_max_ = 0.996
3103 measured reflections
2950 independent reflections
1264 reflections with *I* > 2σ(*I*)
*R*_int_ = 0.056
3 standard reflections every 200 reflections  intensity decay: 1%

#### Refinement

*R*[*F* ^2^ > 2σ(*F* ^2^)] = 0.066
*wR*(*F* ^2^) = 0.077
*S* = 1.00
2950 reflections
226 parameters
H-atom parameters constrained
Δρ_max_ = 0.19 e Å^−3^
Δρ_min_ = −0.19 e Å^−3^

### 

Data collection: *CAD-4 EXPRESS* (Enraf–Nonius, 1994 ▶); cell refinement: *CAD-4 EXPRESS*; data reduction: *XCAD4* (Harms & Wocadlo, 1995 ▶); program(s) used to solve structure: *SHELXS97* (Sheldrick, 2008 ▶); program(s) used to refine structure: *SHELXL97* (Sheldrick, 2008 ▶); molecular graphics: *ORTEP-3 for Windows* (Farrugia, 1997 ▶); software used to prepare material for publication: *SHELXL97*.

## Supplementary Material

Crystal structure: contains datablocks I, global. DOI: 10.1107/S1600536810008378/xu2713sup1.cif

Structure factors: contains datablocks I. DOI: 10.1107/S1600536810008378/xu2713Isup2.hkl

Additional supplementary materials:  crystallographic information; 3D view; checkCIF report

## Figures and Tables

**Table 1:** Hydrogen-bond geometry (Å, °)

*D*—H⋯*A*	*D*—H	H⋯*A*	*D*⋯*A*	*D*—H⋯*A*
N—H0*A*⋯O2^i^	0.86	2.05	2.911 (3)	174

Symmetry code: (i) 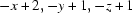.

**Fig. 1.**
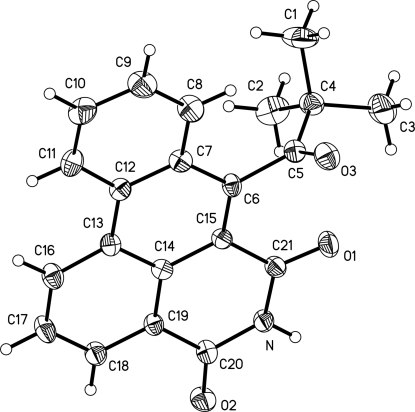
The molecular structure of the title compound with displacement ellipsoids shown at 30% probability ellipsoids for non-H atoms.

## supplementary crystallographic information

### Comment

1,3,4(2*H*)-Isoquinolinetrione derivatives have a variety of biological activities and are synthetic precursors for many naturally occurring alkaloids (Malamas *et al.* 1994; Hall *et al.* 1994). Moreover, many 2-[2'-(dimethyl-amino)ethyl]-1,2-dihydro-3*H*-dibenz[*de*, *h*]- isoquinoline-1,3-dione(azonafide) analogues with structural variations in the side chain and the bent phenanthrene nucleus have shown significant antitumor properties (Sami *et al.* 2000; Hutchings *et al.* 1988). As part of our work involving the synthesis of a series of azonafide analogues from 1,3,4(2*H*)-isoquinolinetrione we report herein the crystal structure of the title compound (Fig. 1).

The carbonyl group forms a dihedral angle of 78.8 (3)° with the phenanthrene moiety. The crystal structure is stabilized by intermolecular π-π stacking; centroids distance between nearly parallel C6-benzene and C17^ii^-benzene rings is 3.851 (2) Å (symmetry code: (ii) 2-x, -y, 1-z]. In addition the intermolecular N—H···O hydrogen bonding further stabilize the crystal structure.

### Experimental

A solution of 1,3,4(2*H*)-isoquinolinetriones (175 mg, 1 mmol) and *tert*-butyl phenyl acetylene (316 mg, 2 mmol) in anhydrous acetonitrile (50 ml) was purged with dry argon for 10 min and then irradiated for 24 h under continuous argon purging. The single crystals of the title compound were obtained from the reaction mixture. The light source was a medium-pressure mercury lamp (500 W) in a cooling water jacket that was further surrounded by a layer of filter solution (1 cm thick, 20% aqueous sodium nitrite) to cut off light of wavelength shorter than 400 nm (Zhang *et al.*, 2000).

### Refinement

H atoms were positioned geometrically and refined using a riding model, with C—H = 0.93 Å for the aromatic atoms, 0.96 Å for the CH_3_ groups and 0.86 Å for the N—H group, and *U*_iso_(H) = 1.2*U*_eq_(C).

### Crystal data

**Table:**

C_21_H_17_NO_3_	*F*(000) = 696
*M**_r_* = 331.36	*D*_x_ = 1.351 Mg m^−^^3^
Monoclinic, *P*2_1_/*c*	Mo *K*α radiation, λ = 0.71073 Å
Hall symbol: -P 2ybc	Cell parameters from 25 reflections
*a* = 11.569 (2) Å	θ = 9–12°
*b* = 9.1150 (18) Å	µ = 0.09 mm^−^^1^
*c* = 15.746 (3) Å	*T* = 298 K
β = 101.12 (3)°	Block, light-yellow
*V* = 1629.3 (6) Å^3^	0.10 × 0.10 × 0.05 mm
*Z* = 4	

### Data collection

**Table:**

Enraf–Nonius CAD-4 diffractometer	1264 reflections with *I* > 2σ(*I*)
Radiation source: fine-focus sealed tube	*R*_int_ = 0.056
graphite	θ_max_ = 25.3°, θ_min_ = 1.8°
ω/2θ scans	*h* = 0→13
Absorption correction: ψ scan (*CAD-4 EXPRESS*; Enraf–Nonius, 1994)	*k* = 0→10
*T*_min_ = 0.991, *T*_max_ = 0.996	*l* = −18→18
3103 measured reflections	3 standard reflections every 200 reflections
2950 independent reflections	intensity decay: 1%

### Refinement

**Table:**

Refinement on *F*^2^	Primary atom site location: structure-invariant direct methods
Least-squares matrix: full	Secondary atom site location: difference Fourier map
*R*[*F*^2^ > 2σ(*F*^2^)] = 0.066	Hydrogen site location: inferred from neighbouring sites
*wR*(*F*^2^) = 0.077	H-atom parameters constrained
*S* = 1.00	*w* = 1/[σ^2^(*F*_o_^2^) + (0.001*P*)^2^] where *P* = (*F*_o_^2^ + 2*F*_c_^2^)/3
2950 reflections	(Δ/σ)_max_ < 0.001
226 parameters	Δρ_max_ = 0.19 e Å^−^^3^
0 restraints	Δρ_min_ = −0.18 e Å^−^^3^

### Special details

**Table:**

Geometry. All e.s.d.'s (except the e.s.d. in the dihedral angle between two l.s. planes) are estimated using the full covariance matrix. The cell e.s.d.'s are taken into account individually in the estimation of e.s.d.'s in distances, angles and torsion angles; correlations between e.s.d.'s in cell parameters are only used when they are defined by crystal symmetry. An approximate (isotropic) treatment of cell e.s.d.'s is used for estimating e.s.d.'s involving l.s. planes.
Refinement. Refinement of *F*^2^ against ALL reflections. The weighted *R*-factor *wR* and goodness of fit *S* are based on *F*^2^, conventional *R*-factors *R* are based on *F*, with *F* set to zero for negative *F*^2^. The threshold expression of *F*^2^ > σ(*F*^2^) is used only for calculating *R*-factors(gt) *etc*. and is not relevant to the choice of reflections for refinement. *R*-factors based on *F*^2^ are statistically about twice as large as those based on *F*, and *R*- factors based on ALL data will be even larger.

### Fractional atomic coordinates and isotropic or equivalent isotropic displacement parameters (Å^2^)

**Table:**

	*x*	*y*	*z*	*U*_iso_*/*U*_eq_	
N	0.9224 (2)	0.3212 (3)	0.52319 (16)	0.0380 (8)	
H0A	0.9433	0.4046	0.5467	0.046*	
O1	0.7998 (2)	−0.0308 (3)	0.72692 (14)	0.0517 (7)	
O2	0.9979 (2)	0.3921 (2)	0.40785 (14)	0.0494 (7)	
O3	0.8470 (2)	0.2652 (2)	0.64021 (14)	0.0508 (7)	
C1	0.5388 (3)	−0.0139 (4)	0.6829 (2)	0.0769 (14)	
H1A	0.5228	−0.0812	0.6352	0.115*	
H1B	0.4670	0.0333	0.6897	0.115*	
H1C	0.5713	−0.0664	0.7349	0.115*	
C2	0.5766 (3)	0.1815 (4)	0.5817 (2)	0.0675 (13)	
H2A	0.5630	0.1123	0.5349	0.101*	
H2B	0.6310	0.2552	0.5704	0.101*	
H2C	0.5035	0.2271	0.5870	0.101*	
C3	0.6483 (3)	0.2081 (4)	0.7420 (2)	0.0781 (15)	
H3A	0.7027	0.2832	0.7326	0.117*	
H3B	0.6803	0.1551	0.7939	0.117*	
H3C	0.5750	0.2524	0.7478	0.117*	
C4	0.6277 (3)	0.1027 (4)	0.6652 (2)	0.0461 (10)	
C5	0.7422 (3)	0.0215 (4)	0.6623 (2)	0.0358 (9)	
C6	0.7748 (3)	−0.0144 (4)	0.5757 (2)	0.0337 (9)	
C7	0.7494 (3)	−0.1610 (4)	0.5410 (2)	0.0358 (9)	
C8	0.7003 (3)	−0.2677 (4)	0.5887 (2)	0.0504 (11)	
H8A	0.6861	−0.2445	0.6433	0.061*	
C9	0.6734 (3)	−0.4046 (4)	0.5553 (3)	0.0584 (12)	
H9A	0.6409	−0.4736	0.5874	0.070*	
C10	0.6942 (3)	−0.4420 (4)	0.4742 (2)	0.0560 (12)	
H10A	0.6738	−0.5346	0.4514	0.067*	
C11	0.7448 (3)	−0.3419 (4)	0.4277 (2)	0.0457 (11)	
H11A	0.7606	−0.3690	0.3742	0.055*	
C12	0.7736 (3)	−0.1988 (4)	0.4589 (2)	0.0351 (9)	
C13	0.8287 (3)	−0.0921 (4)	0.4115 (2)	0.0361 (9)	
C14	0.8587 (3)	0.0469 (4)	0.4492 (2)	0.0314 (9)	
C15	0.8305 (3)	0.0825 (4)	0.5310 (2)	0.0320 (9)	
C16	0.8571 (3)	−0.1202 (4)	0.3306 (2)	0.0430 (10)	
H16A	0.8379	−0.2110	0.3048	0.052*	
C17	0.9120 (3)	−0.0188 (4)	0.2882 (2)	0.0480 (11)	
H17A	0.9299	−0.0415	0.2346	0.058*	
C18	0.9412 (3)	0.1181 (4)	0.3250 (2)	0.0417 (10)	
H18A	0.9782	0.1872	0.2959	0.050*	
C19	0.9154 (3)	0.1513 (4)	0.4046 (2)	0.0324 (9)	
C20	0.9491 (3)	0.2966 (4)	0.4427 (2)	0.0358 (9)	
C21	0.8659 (3)	0.2269 (4)	0.5700 (2)	0.0364 (9)	

### Atomic displacement parameters (Å^2^)

**Table:**

	*U*^11^	*U*^22^	*U*^33^	*U*^12^	*U*^13^	*U*^23^
N	0.052 (2)	0.0271 (19)	0.0377 (18)	−0.0090 (16)	0.0150 (16)	−0.0042 (15)
O1	0.0629 (19)	0.0489 (18)	0.0445 (16)	0.0042 (15)	0.0135 (14)	0.0078 (14)
O2	0.070 (2)	0.0339 (16)	0.0499 (16)	−0.0110 (14)	0.0247 (14)	0.0060 (13)
O3	0.073 (2)	0.0420 (17)	0.0425 (15)	−0.0127 (15)	0.0247 (15)	−0.0123 (13)
C1	0.061 (3)	0.071 (3)	0.109 (4)	−0.017 (3)	0.044 (3)	0.007 (3)
C2	0.058 (3)	0.052 (3)	0.090 (3)	0.008 (2)	0.009 (3)	0.009 (3)
C3	0.079 (3)	0.070 (3)	0.094 (3)	0.002 (3)	0.040 (3)	−0.031 (3)
C4	0.046 (3)	0.041 (3)	0.054 (3)	−0.001 (2)	0.017 (2)	−0.004 (2)
C5	0.050 (3)	0.024 (2)	0.035 (2)	−0.004 (2)	0.011 (2)	0.0057 (18)
C6	0.044 (2)	0.027 (2)	0.030 (2)	0.0027 (19)	0.0083 (18)	−0.0013 (18)
C7	0.044 (3)	0.022 (2)	0.042 (2)	0.0019 (19)	0.010 (2)	0.0068 (19)
C8	0.067 (3)	0.032 (2)	0.056 (3)	0.002 (2)	0.020 (2)	0.005 (2)
C9	0.074 (3)	0.040 (3)	0.067 (3)	−0.005 (3)	0.027 (3)	0.007 (2)
C10	0.069 (3)	0.026 (2)	0.073 (3)	−0.008 (2)	0.014 (3)	0.000 (2)
C11	0.057 (3)	0.027 (2)	0.052 (3)	0.000 (2)	0.005 (2)	−0.007 (2)
C12	0.037 (2)	0.028 (2)	0.040 (2)	0.0029 (19)	0.0064 (19)	−0.0003 (19)
C13	0.039 (2)	0.035 (2)	0.034 (2)	0.002 (2)	0.0067 (19)	−0.0011 (19)
C14	0.032 (2)	0.029 (2)	0.034 (2)	0.0041 (18)	0.0072 (18)	0.0045 (18)
C15	0.036 (2)	0.026 (2)	0.036 (2)	0.0004 (18)	0.0107 (18)	−0.0004 (18)
C16	0.055 (3)	0.034 (2)	0.041 (2)	0.006 (2)	0.014 (2)	−0.004 (2)
C17	0.069 (3)	0.046 (3)	0.033 (2)	0.001 (2)	0.020 (2)	0.000 (2)
C18	0.052 (3)	0.036 (2)	0.039 (2)	−0.003 (2)	0.013 (2)	−0.001 (2)
C19	0.037 (2)	0.027 (2)	0.034 (2)	0.0038 (19)	0.0084 (18)	−0.0022 (18)
C20	0.046 (3)	0.029 (2)	0.033 (2)	0.004 (2)	0.0090 (19)	0.0032 (18)
C21	0.038 (2)	0.037 (2)	0.037 (2)	0.001 (2)	0.0119 (19)	−0.002 (2)

### Geometric parameters (Å, °)

**Table:**

N—C21	1.376 (4)	C7—C12	1.417 (4)
N—C20	1.379 (3)	C8—C9	1.366 (4)
N—H0A	0.8600	C8—H8A	0.9300
O1—C5	1.201 (3)	C9—C10	1.387 (4)
O2—C20	1.224 (3)	C9—H9A	0.9300
O3—C21	1.219 (3)	C10—C11	1.369 (4)
C1—C4	1.541 (4)	C10—H10A	0.9300
C1—H1A	0.9600	C11—C12	1.410 (4)
C1—H1B	0.9600	C11—H11A	0.9300
C1—H1C	0.9600	C12—C13	1.447 (4)
C2—C4	1.514 (4)	C13—C16	1.399 (4)
C2—H2A	0.9600	C13—C14	1.413 (4)
C2—H2B	0.9600	C14—C19	1.416 (4)
C2—H2C	0.9600	C14—C15	1.426 (4)
C3—C4	1.527 (4)	C15—C21	1.476 (4)
C3—H3A	0.9600	C16—C17	1.366 (4)
C3—H3B	0.9600	C16—H16A	0.9300
C3—H3C	0.9600	C17—C18	1.390 (4)
C4—C5	1.526 (4)	C17—H17A	0.9300
C5—C6	1.520 (4)	C18—C19	1.378 (4)
C6—C15	1.365 (4)	C18—H18A	0.9300
C6—C7	1.452 (4)	C19—C20	1.475 (4)
C7—C8	1.413 (4)		
			
C21—N—C20	127.1 (3)	C8—C9—C10	120.8 (4)
C21—N—H0A	116.4	C8—C9—H9A	119.6
C20—N—H0A	116.4	C10—C9—H9A	119.6
C4—C1—H1A	109.5	C11—C10—C9	119.7 (4)
C4—C1—H1B	109.5	C11—C10—H10A	120.1
H1A—C1—H1B	109.5	C9—C10—H10A	120.1
C4—C1—H1C	109.5	C10—C11—C12	121.8 (4)
H1A—C1—H1C	109.5	C10—C11—H11A	119.1
H1B—C1—H1C	109.5	C12—C11—H11A	119.1
C4—C2—H2A	109.5	C11—C12—C7	117.8 (3)
C4—C2—H2B	109.5	C11—C12—C13	122.7 (3)
H2A—C2—H2B	109.5	C7—C12—C13	119.4 (3)
C4—C2—H2C	109.5	C16—C13—C14	117.7 (3)
H2A—C2—H2C	109.5	C16—C13—C12	123.4 (3)
H2B—C2—H2C	109.5	C14—C13—C12	118.9 (3)
C4—C3—H3A	109.5	C13—C14—C19	119.6 (3)
C4—C3—H3B	109.5	C13—C14—C15	120.2 (3)
H3A—C3—H3B	109.5	C19—C14—C15	120.2 (3)
C4—C3—H3C	109.5	C6—C15—C14	122.1 (3)
H3A—C3—H3C	109.5	C6—C15—C21	118.9 (3)
H3B—C3—H3C	109.5	C14—C15—C21	119.0 (3)
C2—C4—C5	113.6 (3)	C17—C16—C13	122.3 (3)
C2—C4—C3	111.3 (3)	C17—C16—H16A	118.8
C5—C4—C3	108.9 (3)	C13—C16—H16A	118.8
C2—C4—C1	108.8 (3)	C16—C17—C18	120.1 (3)
C5—C4—C1	106.5 (3)	C16—C17—H17A	120.0
C3—C4—C1	107.4 (3)	C18—C17—H17A	120.0
O1—C5—C6	118.8 (3)	C19—C18—C17	119.9 (3)
O1—C5—C4	120.7 (3)	C19—C18—H18A	120.0
C6—C5—C4	119.9 (3)	C17—C18—H18A	120.0
C15—C6—C7	118.9 (3)	C18—C19—C14	120.4 (3)
C15—C6—C5	123.1 (3)	C18—C19—C20	118.7 (3)
C7—C6—C5	117.9 (3)	C14—C19—C20	120.9 (3)
C8—C7—C12	119.2 (3)	O2—C20—N	120.0 (3)
C8—C7—C6	120.4 (3)	O2—C20—C19	124.4 (3)
C12—C7—C6	120.4 (3)	N—C20—C19	115.5 (3)
C9—C8—C7	120.6 (4)	O3—C21—N	119.6 (3)
C9—C8—H8A	119.7	O3—C21—C15	123.3 (3)
C7—C8—H8A	119.7	N—C21—C15	117.2 (3)
			
C2—C4—C5—O1	170.9 (3)	C12—C13—C14—C15	−2.5 (5)
C3—C4—C5—O1	46.3 (4)	C7—C6—C15—C14	3.4 (5)
C1—C4—C5—O1	−69.3 (4)	C5—C6—C15—C14	−179.1 (3)
C2—C4—C5—C6	−18.5 (5)	C7—C6—C15—C21	−174.6 (3)
C3—C4—C5—C6	−143.2 (3)	C5—C6—C15—C21	2.9 (5)
C1—C4—C5—C6	101.2 (4)	C13—C14—C15—C6	0.2 (5)
O1—C5—C6—C15	−105.0 (4)	C19—C14—C15—C6	179.3 (3)
C4—C5—C6—C15	84.3 (4)	C13—C14—C15—C21	178.3 (3)
O1—C5—C6—C7	72.5 (4)	C19—C14—C15—C21	−2.7 (5)
C4—C5—C6—C7	−98.2 (4)	C14—C13—C16—C17	0.1 (5)
C15—C6—C7—C8	175.1 (3)	C12—C13—C16—C17	−178.2 (3)
C5—C6—C7—C8	−2.5 (5)	C13—C16—C17—C18	−0.3 (6)
C15—C6—C7—C12	−4.9 (5)	C16—C17—C18—C19	0.4 (5)
C5—C6—C7—C12	177.5 (3)	C17—C18—C19—C14	−0.3 (5)
C12—C7—C8—C9	−1.7 (6)	C17—C18—C19—C20	179.0 (3)
C6—C7—C8—C9	178.3 (4)	C13—C14—C19—C18	0.1 (5)
C7—C8—C9—C10	0.2 (6)	C15—C14—C19—C18	−179.0 (3)
C8—C9—C10—C11	1.6 (6)	C13—C14—C19—C20	−179.2 (3)
C9—C10—C11—C12	−2.0 (6)	C15—C14—C19—C20	1.7 (5)
C10—C11—C12—C7	0.5 (5)	C21—N—C20—O2	178.6 (3)
C10—C11—C12—C13	179.0 (4)	C21—N—C20—C19	−1.6 (5)
C8—C7—C12—C11	1.3 (5)	C18—C19—C20—O2	0.9 (6)
C6—C7—C12—C11	−178.7 (3)	C14—C19—C20—O2	−179.8 (3)
C8—C7—C12—C13	−177.3 (3)	C18—C19—C20—N	−178.9 (3)
C6—C7—C12—C13	2.7 (5)	C14—C19—C20—N	0.4 (5)
C11—C12—C13—C16	0.7 (5)	C20—N—C21—O3	−179.0 (3)
C7—C12—C13—C16	179.3 (3)	C20—N—C21—C15	0.6 (5)
C11—C12—C13—C14	−177.6 (3)	C6—C15—C21—O3	−0.7 (5)
C7—C12—C13—C14	1.0 (5)	C14—C15—C21—O3	−178.8 (3)
C16—C13—C14—C19	0.0 (5)	C6—C15—C21—N	179.6 (3)
C12—C13—C14—C19	178.4 (3)	C14—C15—C21—N	1.6 (5)
C16—C13—C14—C15	179.1 (3)		

### Hydrogen-bond geometry (Å, °)

**Table:**

*D*—H···*A*	*D*—H	H···*A*	*D*···*A*	*D*—H···*A*
N—H0A···O2^i^	0.86	2.05	2.911 (3)	174

Symmetry codes: (i) −*x*+2, −*y*+1, −*z*+1.
